# Dual trajectories of intrinsic capacity and frailty: a joint predictor framework for dementia risk in middle-aged and older adults

**DOI:** 10.1186/s12889-026-28123-4

**Published:** 2026-06-16

**Authors:** Shuanglong Hou, Yi Pan, Xiongxiong Jing, Rui Liu, Jinjin Shen

**Affiliations:** 1 Department of Rehabilitation Medicine, Tangdu Hospital, Fourth Military Medical University, Xi’an, Shaanxi 710038 China; 2 Department of Sport Rehabilitation, Xi’an Physical Education University, Xi’an, Shaanxi 710068 China; 3 Department of Sport Therapy, Shaanxi Provincial Rehabilitation Hospital, Xi’an, Shaanxi 710065 China

**Keywords:** Dementia, Frailty, Group-based dual trajectory modeling, Intrinsic capacity, Joint association

## Abstract

**Background:**

While intrinsic capacity (IC) and frailty are established predictors of adverse health outcomes, their dynamic trajectories and synergistic impact on dementia risk remain underexplored. This study aimed to identify heterogeneous trajectories of IC and frailty across midlife and older age, examine their interrelationships, and determine their independent and joint associations with dementia incidence.

**Methods:**

Data were obtained from the China Health and Retirement Longitudinal Study (CHARLS), with 5,472 participants aged ≥ 50 years followed between 2011 and 2020. We used group-based dual trajectory modeling on data from the 2011–2015 waves to identify distinct IC and frailty trajectories. Incident dementia cases were ascertained at the 2018 and 2020 follow-ups. Cox proportional hazards models evaluated the associations of IC trajectories, frailty trajectories, and their combinations with dementia risk.

**Results:**

Three IC trajectories were identified: stable high, stable moderate, and stable low; and four frailty trajectories: stable robust, improving pre-frail, stable pre-frail, and stable frail. The two sets of trajectories showed strong interdependence. During a median 5-year follow-up, 472 incident dementia cases were recorded. Compared with the stable high IC group, the stable low IC group showed a significantly higher dementia risk (HR: 3.87, 95% CI: 2.62–5.72). Similarly, the stable frail group had an elevated risk (HR: 3.01, 95% CI: 2.15–4.21) relative to the stable robust group. The combination of stable low IC and stable frail trajectories was associated with a 7.31-fold higher risk (HR: 7.31, 95% CI: 3.93–13.59). Incorporating these two trajectories significantly improved the prediction of dementia risk.

**Conclusions:**

Longitudinal IC and frailty trajectories are strong and interdependent predictors of dementia. Dynamic monitoring these trajectories offers a valuable framework for identifying high-risk individuals and guiding targeted interventions to preserve functional capacity, reduce vulnerability, and mitigate dementia risk in aging populations.

**Supplementary Information:**

The online version contains supplementary material available at 10.1186/s12889-026-28123-4.

## Introduction

The accelerating global shift toward aging populations has precipitated a marked rise in dementia prevalence, establishing it as a major public health challenge of the 21st century [1]. Current estimates indicate that over 57 million people worldwide live with dementia, a figure projected to nearly triple by 2050 [2]. Ranked as the eighth leading cause of mortality globally, dementia is associated with staggering economic costs forecast to exceed INT$14,513 billion over the next three decades [3, 4]. Low- and middle-income countries bear a disproportionate burden due to fragile healthcare infrastructures [2], further exacerbated by household financial strain, lost productivity, and caregiver burden [5, 6]. These realities underscore the urgent need for innovative prevention strategies that target modifiable risk pathways across the lifespan. Encouragingly, emerging evidence indicates that a substantial proportion of dementia cases may be preventable, unveiling a promising avenue for mitigating this pressing public health issue [7].

Two complementary constructs—intrinsic capacity (IC) and frailty—have emerged as pivotal biomarkers for predicting dementia risk [8, 9]. IC, as defined by the WHO Integrated Care for Older People (ICOPE) framework, encompasses five functional domains (cognition, locomotion, vitality, sensory, and psychological capacity) and reflects an individual’s dynamic reserve of physical and mental resources [10, 11]. Frailty, in contrast, denotes a state of heightened vulnerability to stressors resulting from the cumulative burden of physiological decline, typically measured through accumulated health deficits [12]. While related and capturing overlapping aspects of biological aging, they represent distinct conceptual models: IC embodies an individual’s functional reservoir [11], whereas frailty quantifies vulnerability to adverse outcomes [12]. Epidemiological evidence confirms that deficits in baseline IC are associated with a 2.17-fold increase in dementia risk [13], while frailty elevates this risk by 1.47-fold [14]; these associations remain robust across diverse populations after adjustment for sociodemographic factors and comorbidities.

Despite the established clinical relevance of IC and frailty, critical knowledge gaps persist. Most prior studies have relied on single-timepoint measurements, failing to capture the dynamic trajectories of these constructs and their differential impact on neurodegeneration. Utilizing a nationally representative Chinese cohort, we employed group-based dual trajectory modeling (GBDTM) to characterize the long-term evolution of IC and frailty. We assessed their independent and joint effects on incident dementia and determined whether these trajectories information enhance dementia risk prediction. The findings are poised to inform the development of targeted, population-level strategies for dementia prevention.

## Materials and methods

### Study population

The China Health and Retirement Longitudinal Study (CHARLS) is an ongoing, nationally representative longitudinal survey designed to collect high-quality microdata from Chinese residents aged ≥ 45 years, supporting interdisciplinary aging and policy research [15]. Employing a multistage, stratified probability-proportionate-to-size (PPS) sampling strategy, CHARLS covers 450 villages/neighborhoods across 150 counties/districts in 28 provinces. The study collects multidimensional data on household structure, health, and socioeconomic factors, with biennial follow-ups beginning in 2011.

For this study, we utilized data from the 2011, 2013, and 2015 waves to model trajectories of IC and frailty. Incident dementia cases were ascertained using 2018 and 2020 wave data. From the initial baseline cohort of 17,708 individuals, we applied sequential exclusion criteria: (1) age < 50 years (*n* = 3,888); (2) missing baseline IC or frailty assessments (*n* = 5,838); (3) missing repeated IC or frailty measures in 2013 and 2015, essential for trajectory modeling (*n* = 1,856); and (4) prevalent dementia at baseline or loss to follow-up during dementia ascertainment (*n* = 654). The final analytical sample comprised 5,472 participants (Figure S1).

### Assessment of intrinsic capacity

IC was defined according to the WHO ICOPE framework [16, 17], a multidimensional construct with established reliability and validity [16, 18]. Five domains were assessed using standardized protocols: (1) Locomotion, evaluated via the Five-Times-Sit-to-Stand test, recording the time to complete five consecutive chair rises; (2) Sensory capacity, based on self-reported vision and hearing status; (3) Psychological capacity, measured with the 10-item Center for Epidemiologic Studies Depression Scale (CESD-10; score range: 0–30). (4) Vitality, captured via handgrip strength, body mass index (BMI), and unintentional weight loss (≥ 5 kg in the past year) [19]; and (5) Cognitive capacity, evaluated with the Telephone Interview for Cognitive Status (TICS, score range 0–31), covering orientation, memory, visuo-construction and attention.

Each domain was ordinally categorized into three levels (0: severe impairment; 1: moderate impairment; 2: fully preserved) [20, 21]. To prevent overlap with the dementia outcome, the cognitive domain was excluded from the composite IC score in the primary analysis, which was calculated as the sum of the remaining four domains (score range: 0–8). Detailed operational definitions, measurement procedures, and scoring criteria are summarized in Table S1.

### Assessment of frailty

Frailty was operationalized using a modified Fried frailty phenotype scale [22], comprising five validated criteria: exhaustion, weakness, low physical activity, weight loss, and slowness. Adapted criteria validated in prior studies [23, 24] were applied as follows: (1) Exhaustion was defined based on two CESD-10 items: whether the participant reported that “I felt everything I did was an effort” or “I could not get going” during the past week [22]. (2) Weakness was identified if participants reported difficulty in lifting or carrying weights over 5 kg [25]. (3) Low physical activity was determined if the participant did not engage in physical activities during a usual week or had not walked continuously for at least 10 min at a time [26]. (4) Weight loss was defined as unintentional weight loss of ≥ 5 kg in the past year or a current BMI < 18.5 kg/m² [27]. (5) Slowness was identified if the participant reported difficulty walking 100 m without resting or climbing several flights of stairs [25].

Each component was dichotomously scored (1 = present; 0 = absent), and a total of frailty score (range: 0–5) was calculated by summing these components, with higher scores indicating greater frailty severity [23, 24].

### Outcome ascertainment

Dementia ascertainment employed dual criteria [28, 29]: (1) an algorithm-based definition requiring concurrent cognitive and functional impairment, consistent with DSM-IV and ICD-10 criteria; or (2) self/caregiver-reported physician-diagnosed dementia or memory-related diseases. The algorithm-based definition has been validated in national surveys [30] and yields prevalence estimates comparable to those in the COAST study and Global Burden of Disease reports. Cognitive function was assessed using the TICS, with domain-specific impairment defined as a score falling 1.5 standard deviations (SDs) below the education-adjusted mean for adults aged ≥ 50. Cognitive impairment was considered present if deficits occurred in at least two domains [31]. Functional impairment was defined as the inability to perform independently at least one basic activities of daily living (including dressing, bathing, eating, transferring, toileting, or continence management), as measured by the Katz-scale [32]. Self-reported cases were identified via the question: “Have you ever been diagnosed with a memory-related disease by a doctor?” This combined methodology has been widely employed in multiple IMPACT-BAM investigations [33, 34] and national prevalence studies [30, 35].

### Covariates

Based on prior studies [28, 29], covariates comprised sociodemographic characteristics, health-related factors, and chronic conditions associated with exposures or dementia outcomes. Sociodemographic variables encompassed age, sex, residence (urban/rural), marital status (married/others), educational attainment (0, 1–6, or > 6 years), and annual per capita household expenditure (tertiles). Health-related factors including drinking (ever/never) and smoking status (ever/never), social participant (yes/no), sleep duration (< 6, 6–8, > 8 h/day), and BMI (< 18.5, 18.5–23.99, ≥ 24.0 kg/m^2^). Chronic conditions were based on self-reported physician diagnoses of non-communicable diseases, including hypertension, dyslipidemia, diabetes, cancer, chronic lung diseases, liver disease, heart attack, stroke, kidney disease, digestive diseases, psychological disorders, arthritis, and asthma. Social participation was defined as engagement in at least one of the following activities during the past month: visiting or spending time with friends, playing mahjong or chess, providing unpaid help to others, going to a park or other public space, participating in community club activities, taking part in volunteer work, attending classes or training courses, and other social activities.

### Statistical analysis

Continuous variables are presented as mean ± SD and categorical variables as frequencies (percentages). Group comparisons utilized one-way ANOVA or χ² tests, as appropriate. Given the 12.74% missing rate in household expenditure (*n* = 697), multiple imputation by chained equations (MICE) was performed.

We employed group-based dual trajectory modeling (GBDTM) to characterize the heterogeneous co-evolution of IC and frailty. This person-centered approach explicitly accounts for population heterogeneity in aging, recognizing that individuals follow distinct developmental pathways rather than uniform, population-averaged trends. Unlike conventional methods reliant on static baseline measures or mean change scores, GBDTM identifies latent subgroups exhibiting qualitatively distinct longitudinal patterns, thereby capturing the multifaceted nature of aging trajectories. The analysis followed a standard procedure [36]. Univariate trajectory models for IC and frailty were fitted separately using data from 2011, 2013, and 2015, testing 2–6 trajectory groups with different polynomial orders. Model selection prioritized the lowest Bayesian and Akaike Information Criteria (BIC, AIC), average posterior probability (AvePP, ≥ 70%), minimum group size (> 5%), and clinical interpretability [37, 38]. Subsequently, a dual-trajectory model was constructed to characterize the joint development of IC and frailty. The output estimated two sets of conditional probabilities—frailty group membership given IC membership, and IC group membership given frailty group membership—as well as the joint probabilities corresponding to specific trajectory combinations.

Cox proportional hazards models estimated hazard ratios (HRs) and 95% confidence intervals (CIs) for the association between trajectory groups and incident dementia. Follow-up time was calculated from baseline to either dementia diagnosis or censoring. Three nested models were constructed: Model 1 adjusted for age, sex, residence, marital status, educational attainment, and household expenditure; Model 2 further adjusted for smoking, drinking, sleep duration, social participation, and BMI; and Model 3 additionally adjusted for chronic diseases. The proportional hazards assumption was verified using Schoenfeld residuals, with variables violating the assumption addressed via stratification.

Based on the identified trajectories, we constructed 12 mutually exclusive joint-trajectory groups. Using the “stable high IC and stable robust” group as the reference, we calculated HRs and 95% CIs for the remaining 11 groups to determine their association with incident dementia. To qualified the incremental predictive utility of trajectory information, we evaluated four models: (1) a base model incorporating all covariates; (2) addition of IC trajectories; (3) addition of frailty trajectories; and (4) addition of joint trajectories. Predictive improvement was assessed using the area under the receiver operating characteristic curve (AUC), integrated discrimination improvement (IDI), net reclassification improvement (NRI), and decision-curve analysis (DCA) to estimate clinical net benefit across risk thresholds.

To investigate potential effect modification, subgroup analyses were conducted according to age (< 60 years vs. ≥60 years), sex, residence, marital status, education level, and household expenditure (tertiles). To assess robustness, we conducted the following sensitivity analyses: (1) separately analyzing algorithm-defined and self-reported dementia cases to assess potential differences arising from different definitions; (2) repeating the primary analysis using complete cases to evaluate the impact of missing data; (3) calculation of E-values to quantify the potential influence of unmeasured confounding on the observed associations; (4) further adjustment for baseline IC and frailty levels, as well as mutual adjustment for trajectories, in the fully adjusted model; and (5) reconstructing trajectories using the full IC construct (including cognitive capacity) to confirm associations with dementia risk.

All statistical analyses were conducted using SPSS 25.0, Stata 17.0, and R 4.3.0, with statistical significance defined as a two-tailed α < 0.05.

## Results

### Baseline characteristics of the study population

A total of 5,472 participants were included in this study, with a mean age of 60.23 ± 6.76 years. Among them, 2,832 (51.8%) were male, 3,487 (63.7%) resided in rural areas, 4,884 (89.3%) were married, and 1,257 (23.0%) had received no formal education. Smoking and drinking status were reported by 1,853 (33.9%) and 1,915 (35.1%) participants, respectively, while 2,961 (54.1%) reported at least one social activity. The prevalence of chronic conditions ranged from 0.7% (cancer) to 34.0% (arthritis). Detailed baseline characteristics are presented in Table 1.

**Table 1:** Baseline characteristics of study population stratified by IC trajectory

Characteristics	Total	IC trajectory			*P* value
		Stable high IC (*n* = 1,340)	Stable moderate IC (*n* = 3,615)	Stable low IC (*n* = 517)	
Age	60.23 ± 6.76	58.97 ± 6.03	60.26 ± 6.79	63.26 ± 7.35	< 0.001
Sex					< 0.001
Male	2832 (51.8)	842 (62.8)	1805 (49.9)	185 (35.8)	
Female	2640 (48.2)	498 (37.2)	1810 (50.1)	332 (64.2)	
Residence					< 0.001
Rural	3487 (63.7)	726 (54.2)	2376 (65.7)	385 (74.5)	
Urban	1985 (36.3)	614 (45.8)	1239 (35.3)	132 (25.5)	
Marrital status					< 0.001
Married	4884 (89.3)	1257 (93.8)	3195 (88.4)	432 (83.6)	
Others	588 (10.7)	83 (6.2)	420 (11.6)	85 (16.4)	
Educational attainment					< 0.001
0 year	1257 (23.0)	184 (13.7)	880 (24.3)	193 (37.3)	
1–6 years	2523 (46.1)	546 (40.7)	1736 (48.0)	241 (46.6)	
>6 years	1692 (30.9)	610 (45.6)	999 (27.6)	83 (16.1)	
Household expenditure					< 0.001
Tertiles 1	1832 (33.5)	373 (27.8)	1234 (34.1)	225 (43.5)	
Tertiles 2	1824 (33.3)	443 (33.1)	1205 (33.3)	176 (34.0)	
Tertiles 3	1816 (33.2)	524 (39.1)	1176 (32.6)	116 (22.5)	
Smoking status					< 0.001
Never	3619 (66.1)	828 (61.8)	2411 (66.7)	380 (73.5)	
Ever	1853 (33.9)	512 (38.2)	1204 (33.3)	137 (26.5)	
Drinking status					< 0.001
Never	3553 (64.9)	768 (57.3)	2383 (65.9)	402 (77.8)	
Ever	1919 (35.1)	572 (42.7)	1232 (34.1)	115 (22.2)	
BMI, kg/m^2^					< 0.001
<18.5	315 (5.8)	27 (2.0)	223 (6.2)	65 (12.6)	
18.5-23.99	2982 (54.5)	722 (53.9)	1985 (54.9)	275 (53.2)	
≥24.0	2175 (39.7)	591 (44.1)	1407 (38.9)	177 (34.2)	
Sleeping time, hours/day					< 0.001
<6	1620 (29.6)	240 (17.9)	1119 (31.0)	261 (50.5)	
6–8	3451 (63.1)	997 (74.4)	2220 (61.4)	234 (45.3)	
>8	401 (7.3)	103 (7.7)	276 (7.6)	22 (4.3)	
Social participants					0.160
Yes	2961 (54.1)	698 (52.1)	1989 (55.0)	274 (53.0)	
No	2511 (45.9)	642 (47.9)	1626 (45.0)	243 (47.0)	
Chronic disease					
Hypertension	1420 (26.0)	283 (21.1)	962 (26.6)	175 (33.8)	< 0.001
Dyslipidemia	548 (10.0)	129 (9.6)	368 (10.2)	51 (9.9)	0.012
Diabetes	347 (6.3)	62 (4.6)	248 (6.9)	37 (7.2)	0.012
Cancer	36 (0.7)	12 (0.9)	21 (0.6)	3 (0.6)	0.465
Chronic lung diseases	544 (9.9)	92 (6.9)	358 (9.9)	94 (18.2)	< 0.001
Liver disease	180 (3.3)	34 (2.5)	125 (3.5)	21 (4.1)	0.159
Heart attack	645 (11.8)	125 (9.3)	413 (11.4)	107 (20.7)	< 0.001
Stroke	99 (1.8)	8 (0.6)	66 (1.8)	25 (4.8)	< 0.001
Kidney disease	315 (5.8)	42 (3.1)	217 (6.0)	56 (10.8)	< 0.001
Digestive diseases	1214 (22.2)	200 (14.9)	839 (23.1)	178 (34.4)	< 0.001
Psychology disease	56 (1.0)	3 (0.2)	40 (1.1)	13 (2.5)	< 0.001
Arthritis	1860 (34.0)	275 (20.5)	1298 (35.9)	287 (55.5)	< 0.001
Asthma	245 (4.5)	37 (2.8)	162 (4.5)	46 (8.9)	< 0.001

*BMI* body mass index, *IC* intrinsic capacity

### Intrinsic capacity and frailty trajectories and associated baseline characteristics

Longitudinal trajectories of IC and frailty were independently modelled, and models specifying 2–6 latent classes were fitted and compared to optimize model fit and interpretability (Table S2). For IC, a three-trajectory model provided the optimal balance between model fit, class interpretability, and classification accuracy, delineating predominantly stable patterns: stable high IC (26.52%), stable moderate IC (61.37%), and stable low IC (12.11%). Similarly, the optimal model comprised four trajectories: stable robust (25.48%), improving pre-frail (8.85%), stable pre-frail (48.07%), and stable frail (17.60%). Classification accuracy was high for both IC (AvePP: 80.21%–83.06%) and frailty (AvePP: 72.12%–79.85%) models.

**Fig. 1:**
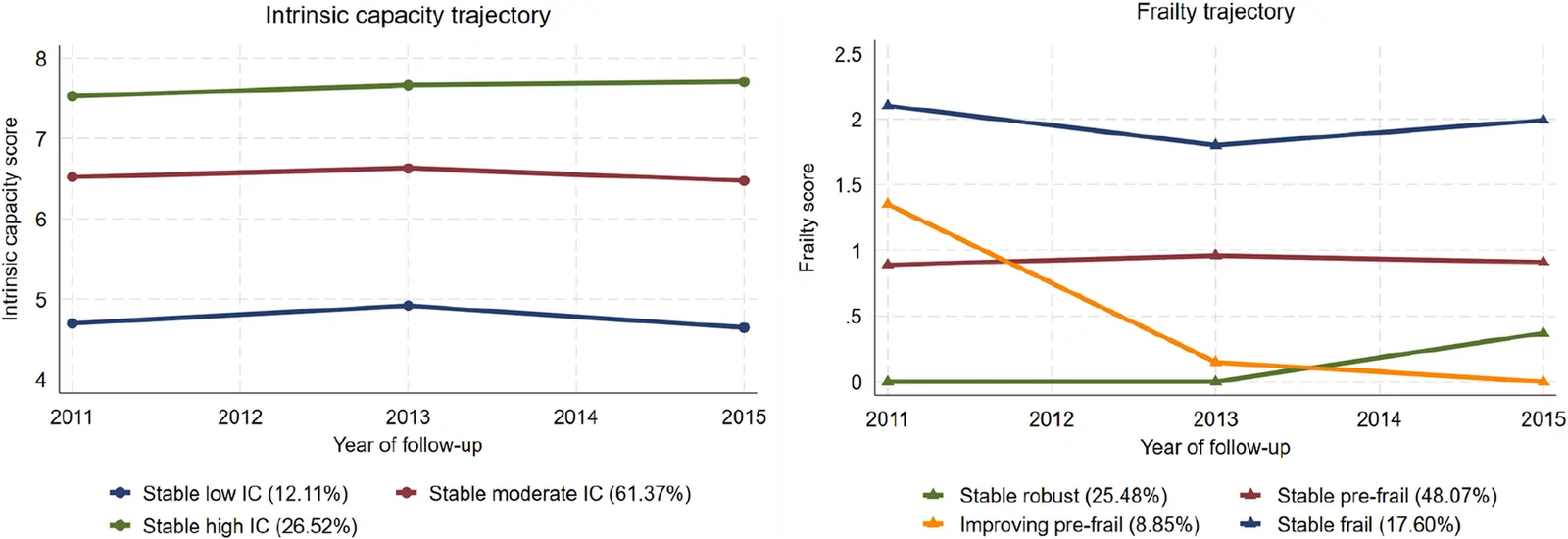
Intrinsic capacity and frailty trajectories. IC, intrinsic capacity

Figure 1 delineates these longitudinal trajectories, revealing predominantly stable patterns across the observation period. Although the relatively flat slopes suggested short-to-medium-term stability within this cohort, between-group differentiation was pronounced. Absolute IC and frailty scores were markedly distinct across trajectories at every assessment wave (Table 2), substantiating the heterogeneity of the identified trajectories. Additionally, distinct trajectories exhibited significant difference in sociodemographic characteristics and baseline health status (Table 1 and S3). Compared with participants in favorable trajectories, those in lower IC or higher frailty trajectories were generally older, reported lower average household expenditure, and more likely to be female, unmarried, or rural residents with insufficient sleep. Furthermore, these participants also had a significantly higher burden of chronic disease, suggesting the clinical relevance of these trajectory-based classifications.

**Table 2:** Scores of intrinsic capacity or frailty across trajectories in 2011, 2013, and 2015

Trajectories	Year of follow-up		
	2011	2013	2015
IC trajectory, IC score			
Stable high IC	7.62 ± 0.59	7.77 ± 0.50	7.89 ± 0.32
Stable moderate IC	6.49 ± 1.11	6.61 ± 1.01	6.43 ± 1.06
Stable low IC	4.33 ± 1.08	4.59 ± 1.24	4.28 ± 1.25
Frailty trajectory, frailty score			
Stable robust	0.00 ± 0.00	0.00 ± 0.00	0.37 ± 0.59
Improving pre-frail	1.35 ± 0.58	0.15 ± 0.03	0.00 ± 0.00
Stable pre-frail	0.89 ± 0.76	0.96 ± 0.65	0.91 ± 0.75
Stable frail	2.10 ± 0.79	1.80 ± 0.71	1.99 ± 0.73

*IC* Intrinsic capacity

### Dual-trajectories of intrinsic capacity and frailty

As summarized in Table 3, dual-trajectory modeling revealed a structured, non-random interdependence between IC and frailty trajectories. Conditioned on IC trajectory membership, individuals with stable high IC exhibited lower frailty burden, with nearly half maintaining robustness (49.25%), whereas those with stable low IC were overwhelmingly belonged to the stable frailty group (64.41%). This relationship proved bidirectional: participants in the stable frail trajectory were largely concentrated in the stable moderate trajectory (61.99%), while those in the stable robust trajectory were predominantly in the stable high IC group (47.35%). Joint probability distribution identified a predominant coupling of specific trajectories, wherein the convergence of stable moderate IC with either stable pre-frail or stable robust states constituted the most prevalent combinations. In contrast, pairings representing the functional extremes, specifically stable low IC with stable robust and improving pre-frail, demonstrated negligible joint probabilities. Logistic regression model further corroborated this robust interdependence (Table S4), lower IC levels correlated significantly with increased odds of belonging to adverse frailty trajectory.

**Table 3:** The interrelationship between intrinsic capacity trajectory and frailty trajectory

IC trajectories	Frailty trajectories			
	Stable robust	Improving pre-frail	Stable pre-frail	Stable frail
A. Probability of frailty trajectories membership given IC trajectories (every row sum to 100%)				
Stable high IC	49.25%	11.42%	36.87%	2.46%
Stable moderate IC	19.97%	8.74%	54.78%	16.51%
Stable low IC	2.32%	2.90%	30.37%	64.41%
B. Probability of IC trajectories membership given frailty trajectories (every column sum to 100%)				
Stable high IC	47.35%	31.61%	18.78%	3.43%
Stable moderate IC	51.79%	65.29%	75.26%	61.99%
Stable low IC	0.86%	3.10%	5.96%	34.58%
C. Joint probability of IC and frailty trajectory membership (all rows and columns sum to 100%)				
Stable high IC	12.06%	2.80%	9.03%	0.61%
Stable moderate IC	13.19%	5.77%	36.18%	10.91%
Stable low IC	0.22%	0.27%	2.87%	6.09%

### Independent associations of intrinsic capacity and frailty trajectories with dementia risk

During the follow-up period, a total of 472 dementia cases were recorded, including 360 self-reported cases and 128 algorithm-identified cases. Table 4 presents the independent associations of distinct IC and frailty trajectories with dementia risk. Compared with the stable high IC reference, both the stable moderate and stable low IC trajectories were associated with a significantly elevated dementia risk. In the fully adjusted model, the HRs were 2.43 (95% CI: 1.75–3.37) for the stable moderate IC and 3.87 (95% CI: 2.62–5.72) for the stable low IC. Similarly, relative to the stable robust trajectory, poorer frailty phenotypes conferred incrementally higher risk. The strongest association was observed for the stable frail trajectory (HR: 3.01, 95% CI: 2.15–4.21), followed by the stable pre-frail trajectory (HR: 1.86, 95% CI: 1.37–2.52). In contrast, the improving pre-frail trajectory did not significantly alter dementia risk (HR: 1.35, 95% CI: 0.86–2.12).

**Table 4:** Association of intrinsic capacity and frailty trajectories with dementia risk

	Model 1		Model 2		Model 3	
	HR (95% CI)	*P* value	HR (95% CI)	*P* value	HR (95% CI)	*P* value
IC trajectory						
Stable high IC	Reference		Reference		Reference	
Stable moderate IC	2.71 (1.96, 3.75)	< 0.001	2.70 (1.95, 3.75)	< 0.001	2.43 (1.75, 3.37)	< 0.001
Stable low IC	5.06 (3.48, 7.36)	< 0.001	5.02 (3.43, 7.34)	< 0.001	3.87 (2.62, 5.72)	< 0.001
Frailty trajectory						
Stable robust	Reference		Reference		Reference	
Improving pre-frail	1.50 (0.96, 2.36)	0.077	1.49 (0.95, 2.36)	0.086	1.35 (0.86, 2.12)	0.192
Stable pre-frail	2.08 (1.54, 2.81)	< 0.001	2.06 (1.52, 2.78)	< 0.001	1.86 (1.37, 2.52)	< 0.001
Stable frail	3.93 (2.86, 5.40)	< 0.001	3.82 (2.77, 5.28)	< 0.001	3.01 (2.15, 4.21)	< 0.001

Model 1 was adjusted for age, sex, residence, marital status, educational attainment, and household expenditure; Model 2 further adjusted for drinking, smoking status, sleep duration, social participant, and BMI; and Model 3 additionally adjusted for hypertension, dyslipidemia, diabetes, cancer, chronic lung diseases, liver disease, heart attack, stroke, kidney disease, digestive diseases, arthritis, and asthma

*IC* intrinsic capacity

### Joint associations of intrinsic capacity and frailty trajectories with dementia risk

**Fig. 2:**
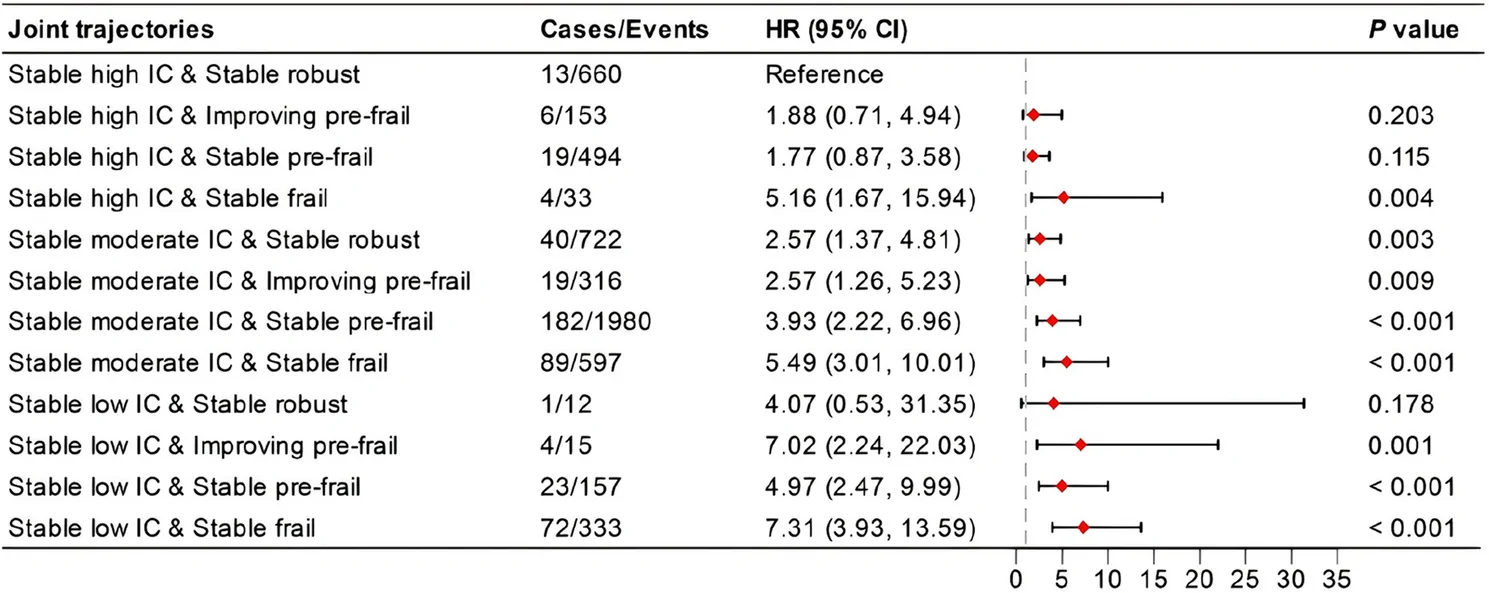
Joint associations of intrinsic capacity and frailty trajectories on dementia risk. Model 1 was adjusted for age, sex, residence, marital status, educational attainment, and household expenditure; Model 2 further adjusted for drinking, smoking status, sleep duration, social participant, and BMI; and Model 3 additionally adjusted for hypertension, dyslipidemia, diabetes, cancer, chronic lung diseases, liver disease, heart attack, stroke, kidney disease, digestive diseases, arthritis, and asthma

Figure 2 presents the joint associations of IC and frailty trajectories with dementia risk. Using the stable high IC and stable robust group as the reference, adjusted HRs demonstrated a graded increase in risk. The highest risk was observed for the combination of stable low IC and stable frail (HR: 7.31, 95% CI: 3.93–13.59), followed by stable low IC with improving pre-frailty (HR: 7.02, 95% CI: 2.24–22.03) and stable moderate IC with stable frail (HR: 5.49, 95% CI: 3.01–10.01). Individuals with stable low IC and stable pre-frailty also showed elevated risk (HR: 4.97, 95% CI: 2.47–9.99). In contrast, the stable high IC with improving pre-frail group (HR: 1.88, 95% CI: 0.71–4.94) and stable pre-frail (HR: 1.77, 95% CI: 0.87–3.58) did not show a significant increase in risk.

### Incremental predictive performance of trajectory information for dementia risk

**Fig. 3:**
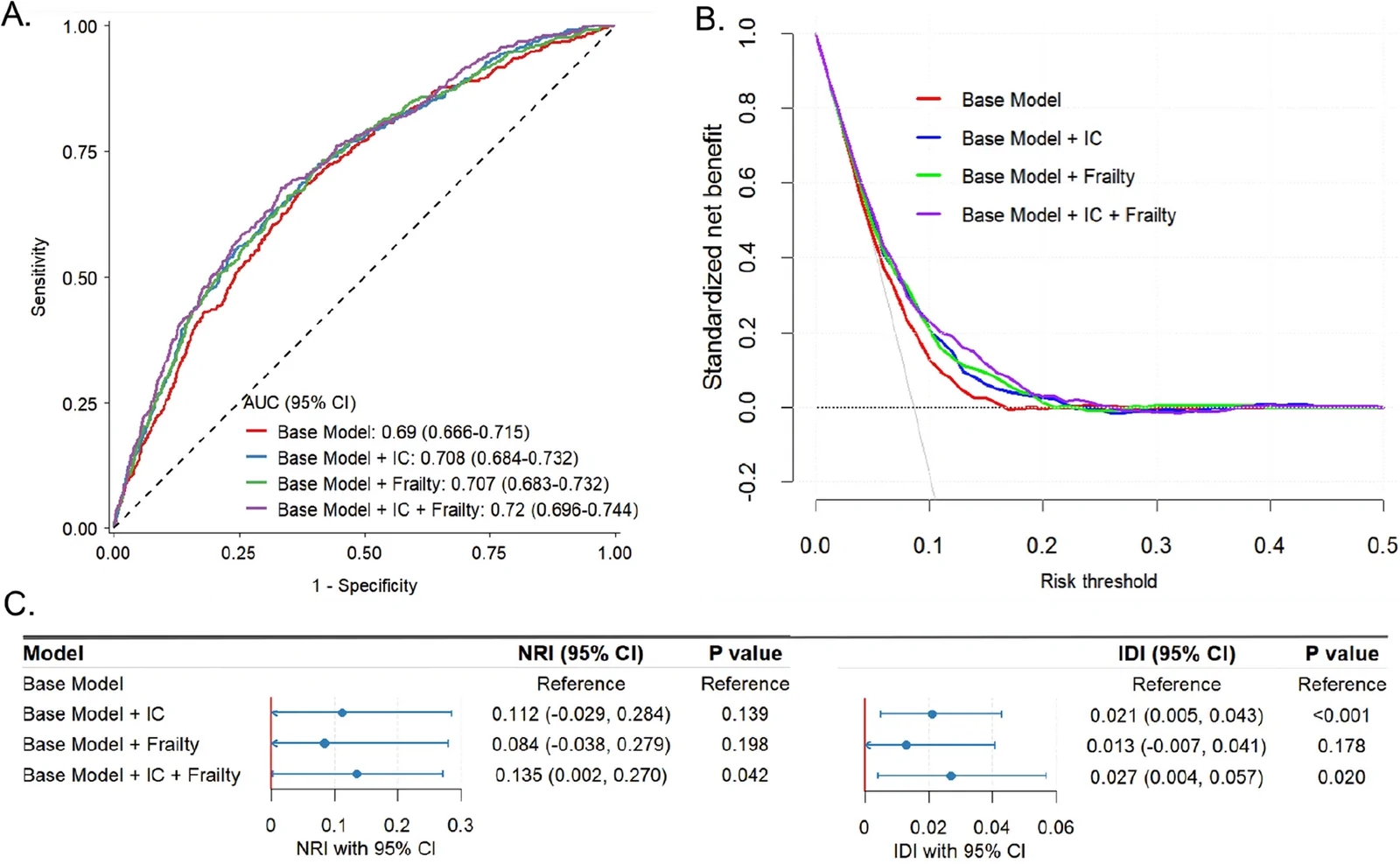
Incremental predictive performance of intrinsic capacity and frailty trajectories for dementia risk. IC, intrinsic capacity; AUC, area under the receiver operating characteristic curve; NRI, net reclassification index; IDI, integrated discrimination improvement. **A** The receiver operating characteristic curves; **B** Decision curve analysis; **C** NRI and IDI indices for intrinsic capacity and frailty trajectories

Figure 3 shows the incremental performance of IC and frailty trajectories for dementia prediction. While the baseline model (including sociodemographic characteristics, health-related lifestyles, and chronic diseases) yielded an AUC of 0.690 (95% CI: 0.666–0.715), adding IC or frailty trajectories alone yielded comparable improvements (AUCs: 0.708 and 0.707, respectively). The integrated model incorporating both trajectories achieved the highest performance (AUC: 0.720, 95% CI: 0.696–0.744; Fig. 3A). Decision curve analysis confirmed superior clinical utility, demonstrating greater net benefit across relevant threshold probabilities (Fig. 3B). Furthermore, inclusion of both trajectories significantly improved risk reclassification, with a NRI of 0.135 (*P* = 0.042) and an IDI of 0.027 (*P* = 0.020) (Fig. 3C).

### Subgroup and sensitivity analyses

Subgroup analyses showed consistent HRs for the association of IC and frailty trajectories with dementia risk, with no significant effect modification (all interaction *P* > 0.05; Tables S5–S6). Sensitivity analyses employing alternative dementia definitions and complete-case datasets yielded results consistent with the main findings (Tables S7–S8). E‑value analysis further suggested robustness of the findings to potential unmeasured confounding (Table S9). Moreover, the associations of IC and frailty trajectories with dementia risk persisted after additional adjustment for baseline IC/frailty levels, and after mutually adjusting for IC and frailty trajectories (Table S10). Finally, IC trajectories constructed with the inclusion of cognitive performance identified three distinct trajectory groups (Figure S2). Using the stable high IC as the reference, the associations between dementia risk and both the moderate IC trajectory and the descending low IC trajectory remained consistent with the primary analysis (Table S11).

## Discussion

This study utilized a nationally representative longitudinal cohort to simultaneously model dynamic changes in IC and frailty, elucidating their interplay in dementia risk. We identified heterogeneous developmental trajectories for both constructs, with dual-trajectory analysis revealing a non-random interdependence between them. Importantly, even after adjusting for extensive confounders and baseline levels, adverse IC and frailty trajectories were independently and jointly associated with elevated dementia risk. Incorporating IC and frailty trajectories into a basic prediction model significantly improved dementia risk prediction. These findings elucidate the intertwined roles of IC and frailty in shaping dementia risk, with important implications for clinical practice and public health strategies.

Our identification of three IC and four frailty trajectories confirm population-level heterogeneity in aging, aligning with the notion that individuals of the same chronological age exhibit divergent biological aging rates [39]. Trajectories such as the stable low IC group characterized by persistently diminished functional reserves, and the stable pre-frail group, marked by a consistent accumulation of health deficits represent subpopulations with high vulnerability, which is consistent with previous research [40–42]. The dual-trajectory model demonstrated clear interdependence: poorer IC trajectories strongly correlated with worse frailty trajectories, and vice versa, empirically supporting their conceptual overlap [43]. While IC captures positive functional capacity and frailty represents deficit accumulation, our findings reinforce their status as complementary facets of biological aging. This interdependence suggests interventions preserving IC may concurrently mitigate frailty [44, 45], and vice versa.

To our knowledge, this is one of the first studies to link longitudinal IC trajectories to dementia risk. Our findings extend prior evidence establishing baseline IC deficits as a risk factor for incident dementia [13]. Employing advanced trajectory modeling, we captured longitudinal IC dynamics—an aspect frequently neglected in prior research. The robust association between adverse trajectories and increased dementia risk, even after adjusting for baseline levels, underscores the importance of monitoring longitudinal trajectories over cross-sectional assessments. These associations are biologically plausible: sustained high IC may bolster cognitive reserve by promoting neurogenesis and reducing neuroinflammation [46, 47], whereas persistent impairment may accelerate neurodegeneration through pathways of mitochondrial dysfunction, oxidative stress, and chronic inflammation [48]. Additionally, IC may directly capture early prodromal dementia symptoms (e.g., depressive symptoms, cognitive decline, hearing loss) [7, 49, 50], thereby predicting subsequent dementia onset.

Our study reinforces the link between dynamic frailty progression and dementia risk, consistent with prior cohort studies [8, 51]. For instance, multinational studies have documented an acceleration in frailty trajectories beginning 4–9 years before clinical dementia diagnosis [51]. Earlier studies further suggest physical frailty may diminish an individual’s physiological reserve, resulting the brain more susceptible to clinical dementia manifestations even amid low-level neuropathology [52]. Progressive frailty indicates accelerated biological aging and compromised capacity to withstand and repair endogenous and environmental damage, heightening neurodegeneration susceptibility [53]. Notably, consistent with Wang et al. [8], reversal of pre-frailty demonstrated no significant association with dementia risk, implying interventions targeting such reversal may yield cognitive benefits. This aligns with the life-course model of dementia prevention, which emphasizes mid- to late-life intervention potential for risk modification [7].

Joint trajectory analysis demonstrated that the coexistence of stable low IC and stable frailty conferred the highest dementia risk (exceeding sevenfold), epitomizing a dual-risk state that overwhelms cerebral compensatory mechanisms. The significant enhancement in risk stratification afforded by integrating these trajectories into conventional models underscores their potential for translation into primary care screening and personalized prevention strategies. From a public health perspective, our findings reinforce the WHO’s emphasis on maintaining IC as a cornerstone of healthy aging and highlight the necessity of monitoring functional reserve and health deficit trajectories [11]. Interventions targeting preservation of locomotor, sensory, vitality, and psychological capacity, including structured exercise [44], nutritional optimization [45], and chronic condition management [45], may concurrently attenuate frailty progression and mitigate dementia risk [44]. Notably, the observed reversibility of pre-frailty in subsets identifies middle-to-late life as a critical window for intervention. Future efforts should prioritize the development of scalable, dynamic IC assessments integrated with frailty trajectories to forge a clinically actionable prediction paradigm, ultimately alleviating the global dementia burden.

This study has several limitations. First, dementia ascertainment relied on self- or caregiver-reported diagnosis, or co-occurring functional and cognitive impairment, rather than standardized neuropsychological assessments. While validated [30], this methodology may introduce misclassification bias. Second, despite robustness suggested by E-value analyses, residual confounding from unmeasured variables (e.g., APOEε4 status, granular dietary patterns) cannot be entirely excluded. Third, trajectory modeling relied on only three assessment waves, which failed to capture long-term fluctuations in individual functional status; extended follow-up periods are warranted in future investigations. Fourth, the inability to differentiate dementia subtypes may obscure pathotype-specific associations, particularly given evidence suggesting divergent IC decline patterns across etiologies [54, 55]. Subtype-specific analyses could improve prognostic precision and intervention targeting. Fifth, frailty assessment relied predominantly on self-report rather than combined self-report and performance-based tests. Although the modified Fried frailty phenotype scale was validated [23, 24], this may have introduced measurement error and potentially attenuated the observed association. Sixth, the cognitive domain was excluded from the composite IC score, leading to an underestimation of the association relative to the WHO ICOPE framework; however, sensitivity analyses using a cognition-inclusive model yielded consistent results. Finally, operational definitions for certain core domains, notably vitality, were adapted for feasibility and may not fully mirror the ICOPE protocol. These adaptations should be contextualized when interpreting our findings.

## Conclusions

This study identifies heterogeneous developmental trajectories of IC and frailty among middle-aged and older Chinese adults and reveals their synergistic impact on dementia risk. Adverse trajectories in IC and frailty were independently and jointly associated with an increased risk of dementia. Incorporating dynamic IC and frailty trajectories significantly improved the accuracy of risk stratification, underscoring their utility in precision dementia prevention. These findings emphasize longitudinal monitoring of functional reserve and vulnerability as critical components of dementia risk assessment. In mid-to-late life, targeted interventions to preserve IC and mitigate frailty may serve as an effective strategy to reduce the growing global burden of dementia.

## Supplementary Information

Supplementary Material 1.

## Data Availability

The CHARLS datasets are publicly accessible and can be obtained directly from the study’s official website: [https://charls.pku.edu.cn/en/].

## Abbreviations

AIC: Akaike Information Criterion
AUC: Area Under the Receiver Operating Characteristic Curve
AvePP: Average Posterior Probability
BIC: Bayesian Information Criterion
CHARLS: China Health and Retirement Longitudinal Study
DCA: Decision Curve Analysis
IC: Intrinsic Capacity
ICOPE: Integrated Care for Older People
IDI: Integrated Discrimination Improvement
NRI: Net Reclassification Index
TICS: Telephone Interview for Cognitive Status
