# Supplementary material for: Dual trajectories of intrinsic capacity and frailty: a joint predictor framework for dementia risk in middle-aged and older adults
Source: BMC Public Health. 2026 Jun 16;26:2420. doi: 10.1186/s12889-026-28123-4 (PMC13501583; doi:10.1186/s12889-026-28123-4)
Supplement: Supplementary file 1 [file 12889_2026_28123_MOESM1_ESM.docx]

**Dual trajectories of intrinsic capacity and frailty: a joint predictor framework for dementia risk in middle-aged and older adults**

**Supplementary materials**

**Table S1** Assessment methods and scoring rubrics for intrinsic capacity

| Subdomains | Code | Assessment methods | Assigned value |
| --- | --- | --- | --- |
| Locomotion | QH001–QH007 | The time required to stand up straight and then sit down onto a chair five times as quickly as possible, with arms crossed in front of the chest. | 0 = chair stand time is 16.70 sec or more; 1 = chair stand time is 11.2 to 16.69 sec; 2 = chair stand time is 11.19 sec or less[1]. |
| Cognitive capacity | DC001–DC003,  DC007, DC027, DC019–DC023,  DC025 | Orientation: Assessed across temporal dimensions including year, month, date, day of the week, and current season. Calculation: Serial subtraction of 7 from 100 for five consecutive iterations. Memory: Includes both immediate recall and delayed recall of 10 target words. Executive Function: Reproduction of a diagram depicting two overlapping pentagons. Total Score of Executive Function ranges from 0 to 31 points. | 0 = ≥1.5 standard deviations below the participant mean; 1 = 1.0–1.49 standard deviations below the participant mean; 2 = others [2]. |
| Sensory capacity | DA022, DA034 | (1) Self-reported distant and near vision status; (2) Self-reported hearing status. | Impaired= “poor”; Unimpaired= “fair”, “excellent”, “very good”, “good”. 0=vision and hearing impaired; 1=vision or hearing impaired; 2=vision and hearing unimpaired [3]. |
| Psychological capacity | DC009–DC018 | The CES-D comprises 10 items: 3 = “always”, 2 = “often”, 1 = “sometimes or rarely,” and 0 = “never,” with items 5 through 8 reverse scored. The total score ranges from 0 to 30. | 0 = more than 19 score; 1 = 10–19 score, 2 = less than 10 score [4]. |
| Vitality capacity | QB001–QB006  QI001, QL002, DA047 | (1) Seated with elbows flex ed at 90 degrees, measure maximum hand grip strength; (2) Unintentional weight loss of ≥5 kg in the past year or a current body mass index (BMI) <18.5 kg/m² | Low handgrip= male <28.0 kg, female <18 kg; weight loss=BMI<18.5kg/m^2^, or weight loss of ≥5 kg in the past year; 0=low handgrip and weight loss; 1= low handgrip or weight loss; 2= normal handgrip and weight loss [5]. |

The cutoffs for all subdomains were determined with reference to established studies to ensure the generalizability of our findings. A score of 0 indicates severe impairment in the respective domain, 1 represents moderate impairment, and 2 denotes fully preserved function.

**Table S2** Evaluation Indices for the Fitting Performance of GBTM

| Class | BIC | AIC | Class proportion (%) | Ave PP (%) |
| --- | --- | --- | --- | --- |
| IC |  |  |  |  |
| Traj_2 | -24262.86 | -24237.04 | 43.76-56.24 | 82.31-88.24 |
| **Traj_3** | **-24064.97** | **--24026.24** | **12.11-61.37-26.52** | **80.45-83.06-80.21** |
| Traj_4 | -24001.11 | -23945.16 | 12.95-14.41-61.64-11.00 | 82.62-62.71-85.67-64.49 |
| Traj_5 | -23988.73 | -23915.57 | 1.70-16.16-56.87-9.59-15.68 | 77.43-74.64-81.37-66.34-65.40 |
| Traj_6 | -24010.31 | -23915.63 | 1.70-2.46-54.43-16.17-9.58-15.67 | 78.46-61.05-78.20-74.38-66.31-65.36 |
| Frailty |  |  |  |  |
| Traj_2 | -21166.60 | -21135.85 | 39.33-60.67 | 84.69-91.25 |
| Traj_3 | -21139.05 | -21092.91 | 27.38-37.98-34.65 | 81.69-62.68-73.97 |
| **Traj_4** | **-20909.79** | **-20855.97** | **17.60-8.85-48.08-25.48** | **79.85-74.85-74.82-72.12** |
| Traj_5 | -20783.64 | -20714.44 | 14.50-13.83-12.98-36.83-21.86 | 82.81-81.58-82.49-63.92-74.81 |
| Traj_6 | -20796.55 | -20708.13 | 14.62-13.26-23.23-30.62-12.91-0.37 | 83.49-79.55--74.77-59.58-76.86-84.91 |

BIC, Bayesian Information Criterion; AIC, Akaike Information Criterion; AvePP, Average posterior probability. This table presents the model fit indices for Group-Based Trajectory Models specifying 2 to 6 trajectories for intrinsic capacity IC and frailty, respectively. The **bolded** row for each construct (IC: Traj_3; Frailty: Traj_4) represents the optimal model selected based on a combination of the lowest BIC/AIC, meaningful group proportions (each subgroup comprising >5% of the sample), and AvePP > 70%.

**Table S3** Baseline characteristics of study population stratified by frailty trajectory

| Characteristics | Total | Frailty trajectory | | | | *P* value |
| --- | --- | --- | --- | --- | --- | --- |
|  |  | Stable robust  (n=1394) | Improving pre-frailty (n=484) | Stable pre-frailty (n=2631) | Stable frailty  (n=963) |  |
| Age | 60.23±6.76 | 59.56±6.45 | 59.86±6.76 | 60.18±6.89 | 61.52±6.70 | < 0.001 |
| Sex |  |  |  |  |  | < 0.001 |
| Male | 2832 (51.8) | 906 (65.0) | 298 (61.6) | 1299 (49.4) | 329 (34.2) |  |
| Female | 2640 (48.2) | 488 (35.0) | 186 (38.4) | 1332 (50.6) | 634 (65.8) |  |
| Residence |  |  |  |  |  | < 0.001 |
| Rural | 3487 (63.74) | 772 (55.4) | 298 (61.6) | 1709 (65.0) | 708 (73.5) |  |
| Urban | 1985 (36.3) | 622 (44.6) | 186 (38.4) | 922 (35.0) | 255 (26.5) |  |
| Marrital status |  |  |  |  |  | 0.001 |
| Married | 4884 (89.3) | 1280 (91.8) | 432 (89.2) | 2339 (88.9) | 833 (86.5) |  |
| Others | 588 (10.7) | 114 (8.2) | 52 (10.7) | 292 (11.1) | 130 (13.5) |  |
| Educational attainment |  |  |  |  |  | < 0.001 |
| 0 year | 1257 (23.0) | 216 (15.5) | 98 (20.2) | 619 (23.5) | 324 (33.6) |  |
| 1-6 years | 2523 (46.1) | 571 (41.0) | 213 (44.0) | 1274 (48.4) | 465 (48.3) |  |
| ＞6 years | 1692 (30.9) | 607 (43.5) | 173 (35.7) | 738 (28.1) | 174 (18.1) |  |
| Household expenditure |  |  |  |  |  | < 0.001 |
| Tertiles 1 | 1832 (33.5) | 420 (30.1) | 170 (35.1) | 866 (32.9) | 376 (36.0) |  |
| Tertiles 2 | 1824 (33.3) | 432 (31.0) | 155 (32.0) | 891 (33.9) | 346 (35.9) |  |
| Tertiles 3 | 1816 (33.2) | 542 (38.9) | 159 (32.9) | 874 (33.2) | 241 (25.0) |  |
| Smoking status |  |  |  |  |  | < 0.001 |
| Ever | 3619 (66.1) | 318 (65.7) | 1747 (66.4) | 718 (74.6) | 836 (60.0) |  |
| Never | 1853 (33.9) | 166 (34.3) | 884 (33.6) | 245 (25.4) | 558 (40.0) |  |
| Drinking status |  |  |  |  |  | < 0.001 |
| Never | 3553 (64.9) | 784 (56.2) | 284 (58.7) | 1736 (66.0) | 749 (77.8) |  |
| Ever | 1919 (35.1) | 610 (45.2) | 200 (41.3) | 895 (34.0) | 214 (22.2) |  |
| BMI, kg/m^2^ |  |  |  |  |  | < 0.001 |
| ＜18.5 | 315 (5.8) | 44 (3.2) | 13 (2.7) | 166 (6.3) | 92 (9.6) |  |
| 18.5-23.99 | 2982 (54.5) | 801 (57.5) | 282 (58.3) | 1419 (53.9) | 480 (49.8) |  |
| ≥ 24.0 | 2175 (39.7) | 549 (39.4) | 189 (39.0) | 1046 (39.8) | 391 (40.6) |  |
| Sleeping time, hours/day |  |  |  |  |  | < 0.001 |
| ＜6 | 1620 (29.6) | 291 (20.9) | 130 (26.9) | 768 (29.2) | 431 (44.8) |  |
| 6-8 | 3451 (63.1) | 996 (71.4) | 314 (64.9) | 1663 (63.2) | 478 (49.6) |  |
| ＞8 | 401 (7.3) | 107 (7.7) | 40 (8.3) | 200 (7.6) | 54 (5.6) |  |
| Social participants |  |  |  |  |  | 0.443 |
| Yes | 2961 (54.1) | 734 (52.7) | 275 (56.8) | 1428 (54.3) | 524 (54.4) |  |
| No | 2511 (45.9) | 660 (47.3) | 209 (43.2) | 1203 (45.7) | 439 (45.6) |  |
| Hypertension | 1420 (26.0) | 302 (21.7) | 122 (25.2) | 692 (26.3) | 304 (31.6) | < 0.001 |
| Dyslipidemia | 548 (10.0) | 109 (7.8) | 47 (9.7) | 275 (10.5) | 117 (12.1) | < 0.001 |
| Diabetes | 347 (6.3) | 63 (4.5) | 27 (5.6) | 159 (6.0) | 98 (10.2) | < 0.001 |
| Cancer | 36 (0.7) | 7 (0.5) | 2 (0.4) | 21 (0.8) | 6 (0.6) | 0.621 |
| Chronic lung diseases | 554 (9.9) | 65 (4.7) | 38 (7.9) | 266 (10.1) | 175 (18.2) | < 0.001 |
| Liver disease | 180 (3.3) | 33 (2.4) | 20 (4.1) | 81 (3.1) | 46 (4.8) | 0.008 |
| Heart attack | 645 (11.8) | 104 (7.5) | 53 (11.0) | 292 (11.0) | 196 (20.4) | < 0.001 |
| Stroke | 99 (1.8) | 9 (0.6) | 13 (2.7) | 43 (1.6) | 34 (3.5) | < 0.001 |
| Kidney disease | 315 (5.8) | 49 (3.5) | 27 (5.6) | 147 (5.6) | 92 (9.6) | < 0.001 |
| Digestive diseases | 1214 (22.2) | 197 (14.1) | 92 (19.0) | 594 (22.6) | 331 (34.4) | < 0.001 |
| Psychology disease | 56 (1.0) | 8 (0.6) | 7 (1.4) | 22 (0.8) | 19 (2.0) | 0.004 |
| Arthritis | 1860 (34.0) | 273 (19.6) | 141 (29.1) | 912 (34.7) | 535 (55.5) | < 0.001 |
| Asthma | 245 (4.5) | 20 (1.4) | 15 (3.1) | 118 (4.5) | 92 (9.6) | < 0.001 |

BMI, body mass index.

**Table S4** Associations between frailty trajectory and IC trajectory

| IC trajectories | IC trajectories (reference: Stable high IC) | | | |
| --- | --- | --- | --- | --- |
|  | Stable moderate IC | | Stable low IC | |
|  | OR (95% CI) | *P* value | OR (95% CI) | *P* value |
| Stable robust | Reference |  | Reference |  |
| Improving pre-frailty | 5.53 (4.42, 6.92) | < 0.001 | 18.50 (12.21, 28.02) | < 0.001 |
| Stable pre-frailty | 8.99 (5.18, 15.59) | < 0.001 | 54.35 (28.00, 105.51) | < 0.001 |
| Stable frailty | 20.89 (11.47, 38.03) | < 0.001 | 207.05 (103.73, 413.28) | < 0.001 |

The model was adjusted for age, sex, residence, marital status, educational attainment, household expenditure, drinking status, smoking status, sleep duration, social participant, BMI, hypertension, dyslipidemia, diabetes, cancer, chronic lung diseases, liver disease, heart attack, stroke, kidney disease, digestive diseases, psychology disease, arthritis, asthma.

**Table S5** Subgroup analysis of the association between IC trajectory and dementia risk

| Characteristics | Stable high IC | Stable moderate IC | | Stable low IC | | *P* for interaction |
| --- | --- | --- | --- | --- | --- | --- |
|  |  | HR (95% CI) | *P* value | HR (95% CI) | *P* value |  |
| Age |  |  |  |  |  | 0.316 |
| ＜60 years | Reference | 2.63 (1.57, 4.42) | < 0.001 | 5.11 (2.69, 9.69) | < 0.001 |  |
| ≥ 60 years | Reference | 2.34 (1.53, 3.58) | < 0.001 | 3.45 (2.10, 5.66) | < 0.001 |  |
| Sex |  |  |  |  |  | 0.952 |
| Male | Reference | 2.32 (1.52, 3.52) | < 0.001 | 3.69 (2.14, 6.36) | < 0.001 |  |
| Female | Reference | 2.57 (1.50, 4.45) | < 0.001 | 4.26 (2.35, 7.73) | < 0.001 |  |
| Residence |  |  |  |  |  | 0.332 |
| Rural | Reference | 2.86 (1.82, 4.51) | < 0.001 | 4.37 (2.61, 7.31) | < 0.001 |  |
| Urban | Reference | 1.88 (1.15, 3.06) | 0.012 | 3.52 (1.85, 6.71) | < 0.001 |  |
| Marrital status |  |  |  |  |  | 0.159 |
| Married | Reference | 2.28 (1.62, 3.18) | < 0.001 | 3.93 (2.61, 5.91) | < 0.001 |  |
| Others | Reference | 4.46 (1.06, 18.82) | 0.042 | 5.05 (1.07, 23.93) | 0.041 |  |
| Educational attainment |  |  |  |  |  | 0.190 |
| 0 year | Reference | 1.51 (0.75, 3.04) | 0.254 | 1.97 (0.89, 4.36) | 0.094 |  |
| 1-6 years | Reference | 3.74 (2.07, 6.77) | < 0.001 | 6.87 (3.56, 13.28) | < 0.001 |  |
| ＞6 years | Reference | 1.99 (1.22, 3.26) | 0.006 | 3.58 (1.77, 7.24) | < 0.001 |  |
| Household expenditure |  |  |  |  |  | 0.476 |
| Tertiles 1 | Reference | 2.25 (1.25, 4.06) | 0.007 | 2.84 (1.42, 5.68) | 0.003 |  |
| Tertiles 2 | Reference | 2.64 (1.47, 4.74) | 0.001 | 5.07 (2.62, 9.81) | < 0.001 |  |
| Tertiles 3 | Reference | 2.35 (1.37, 4.04) | 0.002 | 4.15 (2.05, 8.38) | < 0.001 |  |

IC, intrinsic capacity. All models were adjusted for age, sex, residence, marital status, educational attainment, household expenditure, drinking status, smoking status, sleep duration, social participant, BMI, cognitive function, hypertension, dyslipidemia, diabetes, cancer, chronic lung diseases, liver disease, heart attack, stroke, kidney disease, digestive diseases, and arthritis.

**Table S6** Subgroup analysis of the association between frailty trajectory and dementia risk

| Characteristics | Stable robust | Improving pre-frailty | | Stable pre-frailty | | Stable frailty | | *P* for interaction |
| --- | --- | --- | --- | --- | --- | --- | --- | --- |
|  |  | HR (95% CI) | *P* value | HR (95% CI) | *P* value | HR (95% CI) | *P* value |  |
| Age |  |  |  |  |  |  |  | 0.907 |
| ＜60 years | Reference | 1.38 (0.66, 2.89) | 0.394 | 2.05 (1.26, 3.33) | 0.004 | 3.49 (2.01, 6.07) | < 0.001 |  |
| ≥ 60 years | Reference | 1.33 (0.75, 2.37) | 0.335 | 1.81 (1.22, 2.67) | 0.003 | 2.84 (1.86, 4.35) | < 0.001 |  |
| Sex |  |  |  |  |  |  |  | 0.583 |
| Male | Reference | 1.09 (0.61, 1.97) | 0.764 | 1.69 (1.16, 2.48) | 0.007 | 2.89 (1.84, 4.52) | < 0.001 |  |
| Female | Reference | 2.02 (0.97, 4.23) | 0.061 | 2.31 (1.36, 3.92) | 0.002 | 3.63 (2.09, 6.30) | < 0.001 |  |
| Residence |  |  |  |  |  |  |  | 0.482 |
| Rural | Reference | 1.39 (0.78, 2.47) | 0.261 | 1.91 (1.31, 2.87) | 0.001 | 2.88 (1.88, 4.41) | < 0.001 |  |
| Urban | Reference | 1.27 (0.58, 2.60) | 0.595 | 1.71 (1.05, 2.79) | 0.032 | 3.65 (2.09, 6.39) | 0.012 |  |
| Marrital status |  |  |  |  |  |  |  | 0.375 |
| Married | Reference | 1.38 (0.84, 2.26) | 0.209 | 2.02 (1.45, 2.81) | < 0.001 | 3.21 (2.26, 4.64) | < 0.001 |  |
| Others | Reference | 1.08 (0.35, 3.37) | 0.893 | 1.41 (0.52, 2.50) | 0.740 | 2.17 (0.91, 5.19) | 0.082 |  |
| Educational attainment |  |  |  |  |  |  |  | 0.377 |
| 0 year | Reference | 1.25 (0.45, 3.47) | 0.671 | 1.70 (0.86, 3.35) | 0.128 | 2.01 (0.98, 4.13) | 0.056 |  |
| 1-6 years | Reference | 1.83 (0.93, 3.60) | 0.079 | 2.10 (1.30, 3.41) | 0.003 | 3.73 (2.22, 6.29) | < 0.001 |  |
| ＞6 years | Reference | 0.77 (0.43, 2.18) | 0.936 | 1.71 (1.04, 2.82) | 0.033 | 3.02 (2.02, 6.42) | < 0.001 |  |
| Household expenditure |  |  |  |  |  |  |  | 0.638 |
| Tertiles 1 | Reference | 1.05 (0.47, 2.34) | 0.904 | 1.57 (0.95, 2.60) | 0.0822 | 2.14 (1.22, 3.75) | 0.008 |  |
| Tertiles 2 | Reference | 1.87 (0.85, 4.08) | 0.019 | 2.02 (1.15, 3.55) | 0.015 | 3.95 (2.16, 7.21) | < 0.001 |  |
| Tertiles 3 | Reference | 1.32 (0.59, 2.94) | 0.500 | 2.09 (1.24, 3.53) | 0.006 | 3.36 (1.84, 6.13) | 0.002 |  |

IC, intrinsic capacity. All models were adjusted for age, sex, residence, marital status, educational attainment, household expenditure, drinking status, smoking status, sleep duration, social participant, BMI, cognitive function, hypertension, dyslipidemia, diabetes, cancer, chronic lung diseases, liver disease, heart attack, stroke, kidney disease, digestive diseases, and arthritis.

**Table** **S7** Sensitivity analysis: differentiating algorithm-identified cases from self-reported cases

|  | Model 1 | | Model 2 | | Model 3 | |
| --- | --- | --- | --- | --- | --- | --- |
|  | HR (95% CI) | *P* value | HR (95% CI) | *P* value | HR (95% CI) | *P* value |
| **Self-reported cases** |  |  |  |  |  |  |
| **IC trajectory** |  |  |  |  |  |  |
| Stable high IC | Reference |  | Reference |  | Reference |  |
| Stable moderate IC | 2.93 (2.02, 4.25) | < 0.001 | 2.93 (2.02, 4.26) | < 0.001 | 2.64 (1.81, 3.84) | < 0.001 |
| Stable low IC | 5.03 (3.25, 7.77) | < 0.001 | 4.97 (3.20, 7.73) | < 0.001 | 3.88 (2.46, 6.10) | < 0.001 |
| Frailty trajectory |  |  |  |  |  |  |
| **Stable robust** | Reference |  | Reference |  | Reference |  |
| Improving pre-frailty | 1.26 (0.76, 2.08) | < 0.001 | 1.24 (0.79, 2.05) | 0.400 | 1.12 (0.68, 1.86) | 0.658 |
| Stable pre-frailty | 1.86 (1.35, 2.56) | < 0.001 | 1.85 (1.34, 2.54) | < 0.001 | 1.66 (1.20, 2.29) | 0.002 |
| Stable frailty | 3.11 (2.20, 4.39) | < 0.001 | 2.99 (2.10, 4.25) | < 0.001 | 2.31 (1.60, 3.35) | < 0.001 |
| **Algorithm-identified cases** |  |  |  |  |  |  |
| **IC trajectory** |  |  |  |  |  |  |
| Stable high IC | Reference |  | Reference |  | Reference |  |
| Stable moderate IC | 2.03 (1.10, 3.74) | 0.024 | 2.04 (1.10, 3.78) | 0.023 | 0.71 (0.92, 3.19) | 0.090 |
| Stable low IC | 4.71 (2.38, 9.31) | < 0.001 | 4.82 (2.41, 9.64) | < 0.001 | 3.37 (1.65, 6.88) | 0.001 |
| Frailty trajectory |  |  |  |  |  |  |
| **Stable robust** | Reference |  | Reference |  | Reference |  |
| Improving pre-frailty | 3.82 (1.21, 12.04) | < 0.001 | 3.84 (1.22, 12.10) | 0.022 | 3.59 (1.13, 11.34) | 0.030 |
| Stable pre-frailty | 4.77 (1.90, 11.99) | < 0.001 | 4.70 (1.87, 11.81) | 0.001 | 4.34 (1.72, 10.94) | 0.002 |
| Stable frailty | 13.93  (5.53, 35.06) | < 0.001 | 13.95  (5.52, 35.25) | < 0.001 | 11.52  (4.50, 29.51) | < 0.001 |

IC, intrinsic capacity. Model 1 was adjusted for age, sex, residence, marital status, educational attainment, and household expenditure; Model 2 was further adjusted for drinking status, smoking status, sleep duration, social participant, and BMI; Model 3 additionally adjusted for cognitive function, hypertension, dyslipidemia, diabetes, cancer, chronic lung diseases, liver disease, heart attack, stroke, kidney disease, digestive diseases, arthritis, and asthma.

**Table S8** Sensitivity analysis: repeating the analysis using unimputed data

|  | Model 1 | | Model 2 | | Model 3 | |
| --- | --- | --- | --- | --- | --- | --- |
|  | HR (95% CI) | *P* value | HR (95% CI) | *P* value | HR (95% CI) | *P* value |
| **IC trajectory** |  |  |  |  |  |  |
| Stable high IC | Reference |  | Reference |  | Reference |  |
| Stable moderate IC | 2.69 (1.95, 3.72) | < 0.001 | 2.62 (1.86, 3.68) | < 0.001 | 2.32 (1.68, 3.29) | < 0.001 |
| Stable low IC | 5.02 (3.38, 7.31) | < 0.001 | 4.98 (3.22, 7.15) | < 0.001 | 3.78 (2.55, 5.62) | < 0.001 |
| **Frailty trajectory** |  |  |  |  |  |  |
| Stable robust | Reference |  | Reference |  | Reference |  |
| Improving pre-frailty | 1.51 (0.99, 2.32) | 0.071 | 1.49 (0.93, 2.44) | 0.094 | 1.31 (0.81, 2.02) | 0.203 |
| Stable pre-frailty | 2.08 (1.54, 2.81) | < 0.001 | 2.05 (1.49, 2.87) | < 0.001 | 1.81 (1.30, 2.41) | < 0.001 |
| Stable frailty | 3.82 (2.84, 5.39) | < 0.001 | 3.67 (2.52, 5.37) | < 0.001 | 2.98 (2.10, 4.13) | < 0.001 |

IC, intrinsic capacity. Model 1 was adjusted for age, sex, residence, marital status, educational attainment, and household expenditure; Model 2 was further adjusted for drinking status, smoking status, sleep duration, social participant, and BMI; Model 3 additionally adjusted for hypertension, dyslipidemia, diabetes, cancer, chronic lung diseases, liver disease, heart attack, stroke, kidney disease, digestive diseases, arthritis, and asthma.

**Table S9** Sensitivity analysis: E-value for the association of intrinsic capacity and frailty trajectories with dementia

|  | E-values (95% CI) | | |
| --- | --- | --- | --- |
|  | Model 1 | Model 2 | Model 3 |
| **IC trajectory** |  |  |  |
| Stable high IC | Reference | Reference | Reference |
| Stable moderate IC | 4.86 (3.33, NA) | 4.84 (3.31, NA) | 4.29 (2.90, NA) |
| Stable low IC | 9.59 (6.42, NA) | 9.51 (6.32, NA) | 7.20 (4.68, NA) |
| **Frailty trajectory** |  |  |  |
| Stable robust | Reference | Reference | Reference |
| Improving pre-frailty | 2.37 (1.00, NA) | 2.34 (1.00, NA) | 2.04 (1.00, NA) |
| Stable pre-frailty | 3.58 (2.45, NA) | 3.54 (2.41, NA) | 3.12 (2.08, NA) |
| Stable frailty | 3.93 (2.86, NA) | 7.10 (4.98, NA) | 5.47 (3.72, NA) |

IC, intrinsic capacity. Model 1 was adjusted for age, sex, residence, marital status, educational attainment, and household expenditure; Model 2 was further adjusted for drinking status, smoking status, sleep duration, social participant, and BMI; Model 3 additionally adjusted for hypertension, dyslipidemia, diabetes, cancer, chronic lung diseases, liver disease, heart attack, stroke, kidney disease, digestive diseases, arthritis, and asthma.

**Table S10** Sensitivity analysis: Association of intrinsic capacity and frailty trajectories with dementia risk after further adjusted for baseline IC/frailty and IC/frailty trajectory

|  | Model 4 | | Model 5 | |
| --- | --- | --- | --- | --- |
|  | HR (95% CI) | *P* value | HR (95% CI) | *P* value |
| **IC trajectory** |  |  |  |  |
| Stable high IC | Reference |  | Reference |  |
| Stable moderate IC | 2.16 (1.54, 3.04) | < 0.001 | 2.08 (1.49, 2.91) | < 0.001 |
| Stable low IC | 2.79 (1.76, 4.43) | < 0.001 | 2.78 (1.84, 4.19) | < 0.001 |
| **Frailty trajectory** |  |  |  |  |
| Stable robust | Reference |  | Reference |  |
| Improving pre-frailty | 1.12 (0.69, 1.82) | 0.654 | 1.23 (0.78, 1.93) | 0.379 |
| Stable pre-frailty | 1.65 (1.19, 2.28) | 0.003 | 1.59 (1.17, 2.17) | 0.003 |
| Stable frailty | 2.31 (1.51, 3.50) | < 0.001 | 2.28 (1.60, 3.24) | < 0.001 |

IC, intrinsic capacity. Model 4 was adjusted for age, sex, residence, marital status, educational attainment, household expenditure, drinking status, smoking status, sleep duration, social participant, BMI, hypertension, dyslipidemia, diabetes, cancer, chronic lung diseases, liver disease, heart attack, stroke, kidney disease, digestive diseases, psychology disease, arthritis, asthma, and baseline IC/trajectory; Model 5 was adjusted for adjusted for age, sex, residence, marital status, educational attainment, household expenditure, drinking status, smoking status, sleep duration, social participant, BMI, hypertension, dyslipidemia, diabetes, cancer, chronic lung diseases, liver disease, heart attack, stroke, kidney disease, digestive diseases, psychology disease, arthritis, asthma, and IC/frailty trajectory.

**Table S11** Sensitivity analysis: association between IC trajectories (including cognitive function) and risk of dementia.

|  | Model 1 | | Model 2 | | Model 3 | |
| --- | --- | --- | --- | --- | --- | --- |
|  | HR (95% CI) | *P* value | HR (95% CI) | *P* value | HR (95% CI) | *P* value |
| **IC trajectory** |  |  |  |  |  |  |
| Stable high IC | Reference |  | Reference |  | Reference |  |
| Stable moderate IC | 2.53 (1.90, 3.37) | < 0.001 | 2.53 (1.90, 3.38) | < 0.001 | 2.26 (1.69, 3.02) | < 0.001 |
| Descending low IC | 4.67 (3.30, 6.61) | < 0.001 | 4.66 (3.27, 6.64) | < 0.001 | 3.73 (2.59, 5.35) | < 0.001 |

IC, intrinsic capacity. Model 1 was adjusted for age, sex, residence, marital status, educational attainment, and household expenditure; Model 2 was further adjusted for drinking status, smoking status, sleep duration, social participant, and BMI; Model 3 additionally adjusted for hypertension, dyslipidemia, diabetes, cancer, chronic lung diseases, liver disease, heart attack, stroke, kidney disease, digestive diseases, arthritis, and asthma.

**Figure S1** Flow diagram for the study participants

**Figure S2** Intrinsic capacity trajectory including cognitive function

**Reference**

1.	de Fátima Ribeiro Silva C, Ohara DG, Matos AP, Pinto A, Pegorari MS. Short Physical Performance Battery as a Measure of Physical Performance and Mortality Predictor in Older Adults: A Comprehensive Literature Review. Int J Environ Res Public Health. 2021;18(20).

2.	Hu Y, Peng W, Ren R, Wang Y, Wang G. Sarcopenia and mild cognitive impairment among elderly adults: The first longitudinal evidence from CHARLS. J Cachexia Sarcopenia Muscle. 2022;13(6):2944-52.

3.	He J, Tang X, Jiang M, Zheng X. Associations Between Vision and Hearing Impairment and Cardiovascular Diseases: A Longitudinal Cohort of Middle-Aged and Older Adults in China. J Am Heart Assoc. 2024;13(21):e034851.

4.	Guo Z, Chen Y, Koirala B, Li J, Jia Y, Dennison Himmelfarb CR, et al. Intrinsic capacity trajectories and cardiovascular disease incidence among Chinese older adults: a population-based prospective cohort study. BMC Geriatr. 2025;25(1):269.

5.	Yue X, Yuan Q, Zhou R, Wang M. Social isolation, healthy lifestyle, and intrinsic capacity among older adults in China: A longitudinal study. J Nutr Health Aging. 2025;29(8):100583.
